# Yoga for cancer survivors with chemotherapy‐induced peripheral neuropathy: Health‐related quality of life outcomes

**DOI:** 10.1002/cam4.4098

**Published:** 2021-07-02

**Authors:** W. Iris Zhi, Raymond E. Baser, Lillian M. Zhi, Dristi Talukder, Qing S. Li, Tina Paul, Clare Patterson, Lauren Piulson, Christina Seluzicki, Mary L. Galantino, Ting Bao

**Affiliations:** ^1^ Breast Medicine Service Solid Tumor Division Department of Medicine Memorial Sloan Kettering Cancer Center New York NY USA; ^2^ Department of Epidemiology and Biostatistics Memorial Sloan Kettering Cancer Center New York NY USA; ^3^ Ward Melville High School East Setauket NY USA; ^4^ Integrative Medicine Service Department of Medicine Memorial Sloan Kettering Cancer Center New York NY USA; ^5^ School of Health Sciences Stockton University Galloway NJ USA; ^6^ Perelman School of Medicine University of Pennsylvania Philadelphia PA USA; ^7^ University of Witwatersrand Johannesburg South Africa

**Keywords:** breast cancer, chemotherapy, clinical cancer research, gynecological oncology, QOL, quality of life

## Abstract

**Background:**

Yoga is a meditative movement therapy focused on mind‐body awareness. The impact of yoga on health‐related quality of life (HRQOL) outcomes in patients with chemotherapy‐induced peripheral neuropathy (CIPN) is unclear.

**Methods:**

We conducted a pilot randomized wait‐list controlled trial of 8 weeks of yoga (n = 21) versus wait‐list control (n = 20) for CIPN in 41 breast and gynecological cancer survivors with persistent moderate to severe CIPN. HRQOL endpoints were Hospital Anxiety and Depression Scale (HADS), Brief Fatigue Inventory (BFI), and Insomnia Severity Index (ISI). The Treatment Expectancy Scale (TES) was administered at baseline. We estimated mean changes and 95% confidence intervals (CIs) from baseline to weeks 8 and 12 and compared arms using constrained linear mixed models.

**Results:**

At week 8, HADS anxiety scores decreased −1.61 (−2.75, −0.46) in the yoga arm and −0.32 (−1.38, 0.75) points in the wait‐list control arm (*p *= 0.099). At week 12, HADS anxiety scores decreased −1.42 (−2.57, −0.28) in yoga compared to an increase of 0.46 (−0.60, 1.53) in wait‐list control (*p *= 0.017). There were no significant differences in HADS depression, BFI, or ISI scores between yoga and wait‐list control. Baseline TES was significantly higher in yoga than in wait‐list control (14.9 vs. 12.7, *p *= 0.019). TES was not associated with HADS anxiety reduction and HADS anxiety reduction was not associated with CIPN pain reduction.

**Conclusions:**

Yoga may reduce anxiety in patients with CIPN. Future studies are needed to confirm these findings.

**Clinical Trial Registration Number:** ClinicalTrials.gov Identifier: NCT03292328.

## Graphical abstract

The impact of yoga on health‐related quality of life (HRQOL) outcomes in patients with chemotherapy‐induced peripheral neuropathy (CIPN) is unclear. We conducted a pilot randomized wait‐list controlled trial of 8 weeks of yoga versus wait‐list control for CIPN in breast and gynecological cancer survivors with persistent moderate to severe CIPN. Our results showed a trend for yoga to decrease anxiety, but not Hospital Anxiety and Depression Scale depression scores, Brief Fatigue Inventory, or the Insomnia Severity Index, at weeks 8 and 12 compared to wait‐list control; future studies are needed to confirm these findings.

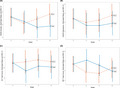

## 1 INTRODUCTION

Chemotherapy‐induced peripheral neuropathy (CIPN) is a common side effect that can persist after chemotherapy completion and is characterized by pain, tingling, numbness, and weakness. CIPN can also interfere with daily functions in patients’ lives and lead to chronic functional decline and lower quality of life. In a study of over 500 cancer survivors, nearly half experienced persistent neuropathy up to 6 years following their completion of chemotherapy treatment.^1^ Presently, the best evidence supporting pharmacological intervention to relieve CIPN symptoms is limited to duloxetine, which is effective for modest pain reduction, but includes potential side effects and undesired drug–drug interactions.^2^ Patients often prefer nonpharmacological integrative approaches, yet more evidence regarding the effectiveness of these approaches is needed.^3^

Yoga is a mind‐body intervention composed of physical and psychological components including postures (asana) and stretching exercises, breathing exercises (pranayama), meditation, relaxation, and ethical guidelines (yamas and niyamas). In patients with cancer diagnosis, benefits of yoga from multiple randomized trials and meta‐analysis include increases in body flexibility and balance, and reductions in stress and anxiety.^4^, ^5^, ^6^ Yoga has also been shown to relieve cancer and treatment‐related symptoms such as nausea, pain, fatigue, and insomnia, and to improve the quality of life in people from different ethnic and language backgrounds.^7^, ^8^, ^9^, ^10^ In patients with CIPN, exercise has been shown to be beneficial for improving functionality, warranting further research of yoga in this specific population.^11^, ^12^, ^13^ Long‐term, persistent CIPN‐associated symptoms not only cause physical dysfunction, such as the risk of falls, but are also associated with psychological distress including anxiety, depression, and insomnia. Fatigue can be both a physical and psychological symptom associated with chronic CIPN. Data regarding if and how yoga alleviates psychological distress associated with CIPN are sparse.

We previously reported on our randomized controlled trial of a yoga intervention for patients with a history of breast or gynecological cancers experiencing persistent moderate to severe CIPN symptoms. Our results showed that yoga reduced CIPN pain and fall risk, and improved physical functioning. Here, we report on health‐related quality of life (HRQOL) outcomes as exploratory secondary endpoints including anxiety, depression, insomnia, and fatigue in these patients experiencing chronic CIPN.

## 2 METHODS

### 2.1 Study participants, design, and intervention

The study participants, randomization, and details of the yoga and wait‐list control intervention have been described previously.^14^ In short, Institutional Review Board approval was attained (ClinicalTrials.gov Identifier: NCT03292328), and eligibility included English‐speaking cancer survivors exposed to neurotoxic chemotherapy, age 18 or older with a primary diagnosis of stage I–III breast, ovarian, or endometrial cancer. We included participants taking pain medication for the past 3 months who could maintain it throughout the study. We excluded those practicing yoga or receiving physical therapy and with metastatic disease.

The yoga group practiced daily for 60 minutes for 8 weeks via video alongside in‐person group classes twice a week. The wait‐list usual care control arm did not receive interventions throughout the 12 weeks. The yoga protocol emphasized breathwork (pranayama) to regulate the autonomic nervous system and modifiable postures (asanas) to improve musculoskeletal flexibility, strength, and balance.

### 2.2 Health‐related quality of life outcomes

We collected HRQOL outcomes including the Hospital Anxiety and Depression Scale (HADS), Brief Fatigue Inventory (BFI), and Insomnia Severity Index (ISI) during the study. We evaluated both yoga and wait‐list control participants at baseline and after weeks 4, 8, and 12. The Treatment Expectancy Scale (TES) was administered at baseline.

#### 2.2.1 Hospital Anxiety and Depression Scale (HADS)

HADS is a self‐report instrument for assessing anxiety and depression symptoms in the past 7 days. HADS is a questionnaire composed of seven anxiety‐related questions and seven depression‐related questions, and has been widely used in cancer populations to assess anxiety and depression severity.^15^ HADS has demonstrated good reliability with a Cronbach's α range of 0.68–0.93; it has an average of 0.83 for anxiety with a range of 0.67–0.9, and an average of 0.83 for depression. HADS also has a good correlation with other similar questionnaires with a range of 0.49–0.83.^15^ Scores of 0–7 are considered not significant, 8–10 are sub‐clinically significant, and 11–21 are indicative of clinically significant depression or anxiety. Participants completed the questionnaire at baseline and after weeks 4, 8, and 12.

#### 2.2.2 Brief Fatigue Inventory (BFI)

The BFI is widely used to measure fatigue in patients with cancer. It has good reliability with a Cronbach's α of 0.96 and a good correlation with other measurements of fatigue (*r* = −0.88, *p *< 0.001).^16^ The BFI items rate the current amount of perceived fatigue a person is experiencing and include the worst and typical fatigue experienced in the prior 24 hrs. Each item is rated on a scale that ranges from 0 (no fatigue) to 10 (as bad as possible). The mean of completed items is used to calculate the BFI score.

#### 2.2.3 Insomnia Severity Index (ISI)

We measured patient‐reported insomnia severity using the ISI in our study. It has a Cronbach's *α* = 0.9 and good validity, especially among patient‐reported outcome measures created to, respectively, evaluate the effect on daytime functioning and level of associated distress.^17^ ISI has also been shown to have internal consistency, specificity, construct validity, and sensitivity.^18^ It has established minimum significant change in value to ensure that the difference is statistically and clinically relevant to patients.^18^ An 8‐point decrease is considered to be a clinically significant improvement.^18^

#### 2.2.4 Treatment Expectancy Scale (TES)

Outcome expectancy can significantly impact treatment outcomes.^19^ TES is a four‐item instrument developed by Mao et al. to assess acupuncture‐related treatment expectancy.^20^ Each item is graded on a 5‐point scale from 0 to 4 and a single total score is calculated (range of 0 to 20), with higher scores indicating greater expectancy. It has demonstrated reliability and validity with a Cronbach's *α* 0.82 and a positive correlation with patient self‐reported efficacy in addition to satisfaction.^20^ TES is also validated among breast cancer survivors and is susceptible to changes in response to acupuncture treatment.^21^ In our previous study, expectancy was shown to be stable in the wait‐list control group and baseline expectancy predicted acupuncture intervention outcomes.^22^ The TES was adapted for use in the current study by replacing the word “acupuncture” with “yoga.”

### 2.3 Statistical analysis

To estimate potential treatment effects and provide insight into symptom trajectories over time while also including patients with missing follow‐up scores in the analysis per the intention‐to‐treat principle, we analyzed each outcome measure using a constrained linear mixed model (cLMM). We constrained the treatment arms to have a common baseline mean,^23^ reflecting the pre‐randomization timing of the baseline assessment. The dependent variable vector included the pre‐randomization baseline (week 0) assessment, as well as all post‐randomization assessments at weeks 4, 8, and 12. The independent variables were treatment arm, week (categorical), and arm‐by‐week interaction. A patient‐level random intercept was included in the model to account for the repeated outcome measurements within patients. All randomized patients with at least one outcome assessment were included in the model. Results are reported as least‐squares means, mean differences, and confidence intervals (CIs), with inferences regarding differences between arms based on model coefficients from the arm‐by‐week interaction and contrasts of model‐adjusted means. We pre‐specified comparisons between arms at 2‐time points of interest, week 8 and week 12. From the cLMM for each outcome, we calculated the model‐based means and 95% CIs by study arm and assessment time and used a series of contrasts to test for significant within‐arm changes from baseline as well as between‐arm differences in changes from baseline. Differences between arms on categorical variables were tested using Fisher's exact tests.

## 3 RESULTS

We have previously described the detailed characteristics of the patients in this study (Table 1).^14^ A total of 41 patients were enrolled and randomized into yoga (n = 21) and usual care (n = 20) arms. Patients were balanced between two arms, although the usual care arm had more patients who received paclitaxel alone chemotherapy compared with the yoga arm (5% vs. 33.3%).

**TABLE 1:** Patient characteristics

Characteristic*	Overall, N = 41	Yoga, N = 21	WLC, N = 20
Patient age	61.7 (35.5, 79.0)	60.0 (35.5, 77.9)	62.3 (42.4, 79.0)
Body mass index	26.6 (17.8, 35.9)	26.6 (18.7, 35.5)	26.5 (17.8, 35.9)
Race			
White	23 (56.1%)	11 (52.4%)	12 (60.0%)
Black	8 (20.0%)	4 (19.0%)	4 (20.0%)
Asian	5 (12.2%)	4 (19.0%)	1 (5.0%)
Unknown	5 (12.2%)	2 (9.5%)	3 (15.0%)
Ethnicity			
Hispanic	2 (4.9%)	1 (4.8%)	1 (5.0%)
Non‐Hispanic	39 (95.1%)	20 (95.2%)	19 (95.0%)
Cancer type			
Breast	38 (92.7%)	18 (85.7%)	20 (100.0%)
Uterine	2 (4.9%)	2 (9.5%)	0 (0%)
Ovary	1 (2.4%)	1 (4.8%)	0 (0%)
Cancer stage			
Stage I	11 (26.8%)	6 (28.6%)	5 (25.0%)
Stage II	15 (36.6%)	5 (23.8%)	10 (50.0%)
Stage III	13 (31.7%)	9 (42.9%)	4 (20.0%)
Other	2 (4.9%)	1 (4.8%)	1 (5.0%)
Years Since Diagnosis	3.9 (0.9, 25.8)	3.5 (0.9, 25.8)	4.1 (1.3, 15.8)
Years Since CTx End	3.1 (0.5, 15.3)	3.1 (0.5, 10.4)	3.7 (0.9, 15.3)
Type of CTx			
Carboplatin	1 (2.4%)	1 (4.8%)	0 (0.0%)
Docetaxel	2 (4.9%)	2 (9.5%)	0 (0.0%)
Docetaxel & Carboplatin	3 (7.3%)	2 (9.5%)	1 (5.0%)
Paclitaxel	33 (80.5%)	14 (66.7%)	19 (95.0%)
Paclitaxel & Carboplatin	2 (4.9%)	2 (9.5%)	0 (0.0%)

Abbreviations: CTx, Chemotherapy; WLC, Wait‐list Control.

* Statistics presented: median (minimum, maximum); n (%).

### 3.1 HADS anxiety

**FIGURE 1:**
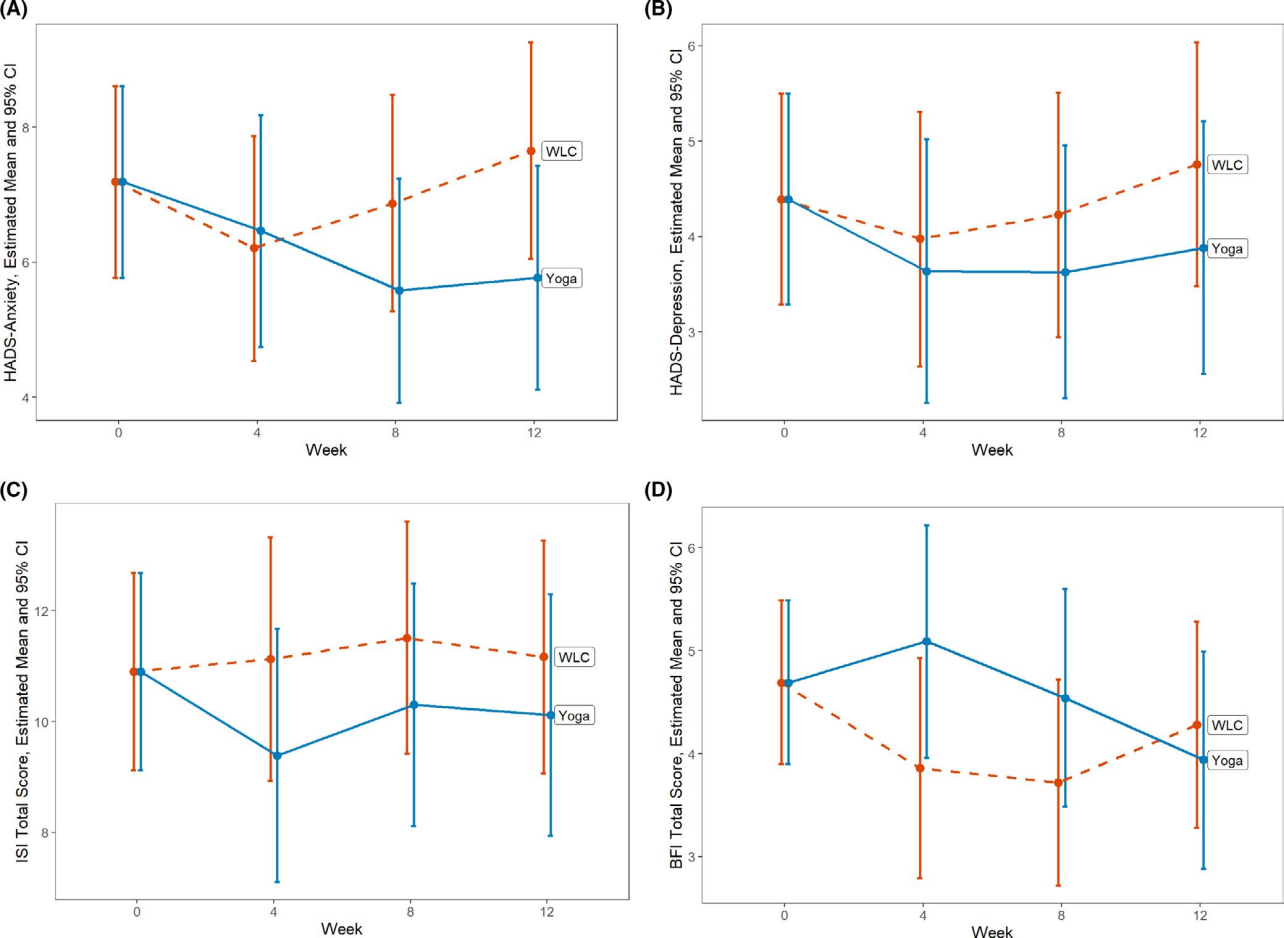
Health‐related quality of life outcome changes by week and treatment arm. Data points represent the model‐estimated means and 95% confidence intervals (indicated by the I bars) from a constrained linear mixed model (cLMM) with baseline means constrained to be equal across study arms, reflecting the pre‐randomization nature of the baseline assessment. See Methods section for model details. A, HADS Anxiety subscale ranges from 0 to 21, with higher scores indicating higher anxiety symptoms. B, HADS Depression subscale ranges from 0 to 21, with higher scores indicating higher depressive symptoms. C, ISI Total score ranges from 0 to 28, with higher scoring indicating higher insomnia symptoms. D, BFI Total score ranges from 0 to 10, with higher scores indicating higher fatigue. Abbreviations: BFI, Brief Fatigue Inventory; CI, confidence interval; HADS, Hospital Anxiety and Depression Scale; ISI, Insomnia Severity Index; WLC, wait‐list control

HADS anxiety scores were reported in both arms at baseline, 4, 8, and 12 weeks (Table 1 and Figure 1A). At baseline, the model‐constrained common mean HADS anxiety score was 7.19 (95% CI 5.77, 8.61) for both arms. At week 8, the HADS anxiety score decreased −1.61 (−2.75, −0.46) points in the yoga arm and −0.32 (−1.38, 0.75) points in the wait‐list control arm, for a difference of −1.29 (−2.83, 0.25) (*p* = 0.099). This difference translates to an effect size, Cohen's d, of d = 0.54. At week 12, relative to baseline the HADS anxiety score decreased −1.42 (−2.57, −0.28) in the yoga arm compared to an increase of 0.46 (−0.60, 1.53) in the wait‐list control arm, for a difference of −1.88 (−3.42, −0.34) (*p* = 0.017). This difference translates to an effect size, Cohen's d, of *d* = 0.62.

Using the predefined HADS anxiety category, at baseline, nine (43%) patients in the yoga arm had clinically significant anxiety (defined as score 11 or higher out of 21).^15^ In comparison, the wait‐list control arm had three (15%) patients with clinically significant anxiety at baseline (*p* = 0.09). At week 12, the yoga arm had three (19%) patients and the wait‐list control arm had four (21%) patients with clinically significant anxiety (*p *> 0.99).

HADS anxiety reduction was not associated with CIPN pain reduction by Pearson correlation analysis at weeks 4, 8, and 12 (*r* = −0.07, −0.16, and 0.02, respectively, with *p* = 0.72, 0.37, and 0.9, respectively).

### 3.2 TES

TES scores were collected in both arms at baseline. The mean (standard deviation, SD) TES score at baseline was 14.9 (3.27) in the yoga arm, which was significantly higher than in the wait‐list control arm 12.7 (2.58), *p* = 0.019. Further analysis revealed that the TES scores were not associated with HADS anxiety reduction by Pearson correlation analysis at weeks 4, 8, and 12 (*r* = 0.23, 0.15, and −0.01, respectively, with *p* = 0.23, 0.38, and 0.99, respectively).

### 3.3 Other outcomes

HADS depression, ISI, and BFI scores were reported at baseline, and at weeks 4, 8, and 12 (Table 1, Figure 1B–D). There was no significant difference in HADS depression, ISI, or BFI scores between yoga and wait‐list control arms at baseline, week 8, and week 12 (Table 2).

**TABLE 2:** Health‐related quality of life outcomes

Outcome	Week	Yoga arm			WLC arm			Differences in change from baseline (Yoga ‐ WLC), Mean (95% CI)	*p*‐value Diffs
		n	Mean (95% CI)	Change from Baseline, Mean (95% CI)	n	Mean (95% CI)	Change from Baseline, Mean (95% CI)		
HADS Anxiety	0	21	7.19 (5.77, 8.61)		20	7.19 (5.77, 8.61)			
	4	13	6.47 (4.75, 8.18)	−0.72 (−1.96, 0.51)	15	6.21 (4.54, 7.87)	−0.98 (−2.14, 0.18)*	0.26 (−1.41, 1.93)	0.759
	8	16	5.58 (3.92, 7.24)	−1.61 (−2.75, −0.46)‡	19	6.87 (5.27, 8.48)	−0.32 (−1.38, 0.75)	−1.29 (−2.83, 0.25)*	0.099
	12	16	5.77 (4.11, 7.43)	−1.42 (−2.57, −0.28)†	19	7.65 (6.05, 9.26)	0.46 (−0.60, 1.53)	−1.88 (−3.42, −0.34)†	0.017
HADS Depression	0	21	4.39 (3.29, 5.50)		20	4.39 (3.29, 5.50)			
	4	13	3.64 (2.26, 5.02)	−0.75 (−1.82, 0.31)	15	3.98 (2.64, 5.31)	−0.41 (−1.42, 0.59)	−0.34 (−1.77, 1.10)	0.643
	8	16	3.63 (2.31, 4.96)	−0.76 (−1.75, 0.23)	19	4.23 (2.95, 5.51)	−0.16 (−1.08, 0.76)	−0.60 (−1.92, 0.73)	0.373
	12	16	3.88 (2.56, 5.21)	−0.51 (−1.50, 0.48)	19	4.76 (3.48, 6.04)	0.37 (−0.55, 1.29)	−0.87 (−2.20, 0.45)	0.194
ISI Total	0	21	10.90 (9.13, 12.68)		20	10.90 (9.13, 12.68)			
	4	13	9.39 (7.11, 11.67)	−1.51 (−3.35, 0.32)	15	11.13 (8.93, 13.32)	0.22 (−1.50, 1.95)	−1.74 (−4.21, 0.74)	0.167
	8	16	10.30 (8.12, 12.49)	−0.60 (−2.30, 1.11)	19	11.51 (9.42, 13.61)	0.61 (−0.98, 2.20)	−1.21 (−3.48, 1.07)	0.295
	12	16	10.12 (7.94, 12.30)	−0.78 (−2.49, 0.92)	19	11.17 (9.07, 13.26)	0.26 (−1.32, 1.85)	−1.05 (−3.32, 1.23)	0.363
BFI Composite	0	20	4.69 (3.90, 5.49)		19	4.69 (3.90, 5.49)			
	4	13	5.09 (3.96, 6.22)	0.40 (−0.66, 1.45)	15	3.86 (2.79, 4.93)	−0.84 (−1.82, 0.15)*	1.24 (−0.15, 2.62)*	0.081
	8	16	4.54 (3.49, 5.60)	−0.15 (−1.11, 0.81)	19	3.72 (2.72, 4.72)	−0.97 (−1.88, −0.07)†	0.82 (−0.44, 2.08)	0.199
	12	16	3.94 (2.88, 4.99)	−0.76 (−1.72, 0.21)	19	4.28 (3.28, 5.28)	−0.41 (−1.32, 0.49)	−0.34 (−1.60, 0.92)	0.591

Abbreviations: BFI, Brief Fatigue Inventory; CI, confidence interval; HADS, Hospital Anxiety and Depression Scale; ISI, Insomnia Severity Scale; WLC, Wait‐list control.

* *p* < 0.1;

† *p* < 0.05;

‡ *p* < 0.01;

*** *p* < 0.001

Pain medication usages were reviewed at baseline, and at weeks 4, 8, and 12. Neither the percentage of patients taking pain medications nor the number of pain medications was significantly different between the yoga and wait‐list control arms throughout the entire study period (Table 3).

**TABLE 3:** Numbers of pain medications

	Number of medications	Overall	Yoga	WLC	*p*‐value
Baseline	0	29 (70.7%)	15 (71.4%)	14 (70.0%)	0.492
	1	10 (24.4%)	6 (28.6%)	4 (20.0%)	
	2	2 (4.9%)	0 (0.0%)	2 (10.0%)	
Week 4	0	27 (81.8%)	16 (84.2%)	11 (78.6%)	0.331
	1	4 (12.1%)	3 (15.8%)	1 (7.1%)	
	2	2 (6.1%)	0 (0.0%)	2 (14.3%)	
Week 8	0	24 (72.7%)	13 (81.2%)	11 (64.7%)	0.517
	1	7 (21.2%)	3 (18.8%)	4 (23.5%)	
	2	2 (6.1%)	0 (0.0%)	2 (11.8%)	
Week 12	0	23 (69.7%)	12 (75.0%)	11 (64.7%)	0.733
	1	6 (18.2%)	2 (12.5%)	4 (23.5%)	
	2	3 (9.1%)	2 (12.5%)	1 (5.9%)	
	3	1 (3.0%)	0 (0.0%)	1 (5.9%)	

Abbreviations: WLC, Wait‐list Control.

## 4 DISCUSSION

In this manuscript, we report the secondary exploratory endpoint in HRQOL outcomes, including HADS anxiety, HADS depression, ISI, BFI, and TES, from a randomized controlled phase II clinical trial comparing a yoga intervention with wait‐list control. The primary results of the trial, which we published previously, showed that yoga reduced CIPN pain by 1.95 points using a numeric rating scale in the yoga arm, but that it was not statistically different from usual care (*p* = 0.14). However, yoga improved the Functional Assessment of Cancer Therapy/Gynecologic Oncology Group‐Neurotoxicity subscale (FACT/GOG‐Ntx) by 4.25 points versus 1.36 points in usual care (*p* = 0.035). Further, it decreased the risk of falls as measured by the Functional Reach Test, which improved by 7.14 cm in the yoga group and decreased by 1.65 cm in usual care (*p* = 0.001). This suggests that yoga improved CIPN‐associated pain and may help reduce the risk of falls.^14^ Here, our results showed a trend for yoga to decrease anxiety, but not HADS depression, BFI, or ISI, at weeks 8 and 12 compared to wait‐list control in this exploratory analysis. The TES was significantly higher in yoga than in wait‐list control, but it was not associated with HADS anxiety reduction. HADS anxiety reduction was not associated with CIPN pain reduction.

It is important to note that HADS anxiety scores were not balanced between yoga (mean = 9.2, SD = 4.1) and wait‐list control (mean = 5.0, SD = 4.0) at baseline. By definition, such baseline imbalances are due to chance in randomized trials. Unfortunately, due to the relatively small sample size and baseline imbalance between the arms, the differences in HADS anxiety score may change over time were due to regression‐to‐the‐mean rather than to the yoga intervention. However, our model‐based inferences are robust to random differences between the arms at baseline because we used the cLMM analysis method to constrain the two study arms to have a common baseline mean, reflecting that the baseline assessment occurred before randomization and that study participants represented a single population at that time point. Additionally, examining the within‐arm changes from baseline to weeks 8 and 12 in Table 2, the yoga arm had significantly improved HADS anxiety scores, with mean (95% CI) improvement of −1.61 (−2.75, −0.46) points at 8 weeks (*p* = 0.006) and −1.42 (−2.57, −0.28) points at 12 weeks (*p* = 0.016). The wait‐list control arm had non‐significant changes, a decrease of −0.32 (−1.38, 0.75) at 8 weeks (*p* = 0.56) and an increase of 0.46 (−0.60, 1.53) at 12 weeks (*p* = 0.39). Although not completely incompatible with the regression‐to‐the‐mean explanation, these patterns of within‐arm changes are more consistent with the conclusion that 8 weeks of biweekly yoga might have helped reduce anxiety symptoms compared to wait‐list control.

The TES was originally developed for acupuncture expectancy and is a significant predictor for acupuncture treatment outcome.^21^ Our result showed that there was a significantly higher TES score in the yoga arm compared to the wait‐list control arm, indicating high treatment expectancy in the yoga arm. However, there was no association between TES and HADS anxiety reduction, suggesting that the effects of yoga on decreasing anxiety were not simply due to high treatment expectancy. Importantly, it is unknown whether the TES for yoga has similar validity and reliability as for acupuncture. However, others have reported conflicting results in expectancy and efficacy in yoga interventions, suggesting the necessity of further developing accurate assessments.^24^, ^25^, ^26^

Our results are consistent with existing literature finding the effectiveness of yoga in reducing anxiety in cancer patients. Banerjee et al. reported a significant 4.4‐point reduction in HADS anxiety and depression scores (on a 21‐point scale) among breast cancer patients undergoing radiotherapy in the yoga intervention group.^27^ Another study found that anxiety in cancer survivors significantly decreased after an 8‐week yoga intervention but increased slightly at the 6‐month follow‐up.^28^ A recent review of yoga in managing cancer and treatment‐related symptoms suggests that yoga demonstrated improved the overall quality of life in patients with cancers during and after anticancer treatments in multiple randomized controlled trials. However, the review found less consistency and mixed results in terms of anxiety, depression, and psychological outcomes.^29^

Several studies have explored the possible molecular mechanism of yoga and anxiety. Elevated serum pro‐inflammatory cytokines and cortisol levels, and reduced brain‐derived neurotrophic factor (BDNF) have been associated with anxiety.^30^, ^31^, ^32^ In a study by Cahn et al., BDNF levels significantly increased after a 3‐month yoga retreat with lower anxiety levels among the participants.^33^ Cortisol levels were measured during, before, and after the yoga intervention without any difference seen. Interestingly, the study also measured a panel of proinflammatory cytokines, including interleukin (IL)‐1b, IL‐6, IL‐8; tumor necrosis factor (TNF‐α); and interferon (INF‐γ). All of these had significantly higher serum concentration after the yoga intervention; however, IL‐10 (anti‐inflammatory cytokine) and IL‐12 (pro‐inflammatory cytokine) decreased after the yoga intervention.^33^ A recent systematic review examining 15 studies of yoga and inflammation markers in variable chronic conditions, including cancer, cardiovascular disease, and autoimmune disease, concluded that yoga might be a viable modality to reduce inflammation depending on yoga dose.^34^ Cortisol, BDNF, and cytokines dynamics have also been studied in depression, fatigue, and other psychological disorders, as they often coexist with anxiety; however, results from these studies are inconsistent and inconclusive.^35^, ^36^, ^37^, ^38^, ^39^

Chronic pain, a symptom of CIPN, may be exacerbated by stress, is associated with low heart rate variability, and has shown improvement in response to yoga‐based interventions.^40^ In a recent systematic review of yoga in geriatric populations, yoga was associated with significant improvement in multiple physical functions and HRQOL outcomes including balance, strength, and flexibility, in addition to mental wellbeing.^41^ Given that cancer survivors tend to be more sedentary,^42^ it is important to assure cardiovascular conditioning when recommending exercise and physical activity for individuals living with and beyond cancer. However, adherence to a physical activity program may be hampered by CIPN, thus yoga may be a viable first option to ease into physical activity and meet these standards. Future studies with detailed descriptions of the specific type and frequency of yoga, assessment of well‐established inflammatory and cardiovascular biomarkers, and larger sample sizes will help advance our knowledge of the impact of yoga on the hypothalamic‐pituitary‐adrenal‐cortical system.

CIPN is associated with impaired quality of life in patients with a cancer diagnosis, which may include psychological distress and sleep disturbance.^43^, ^44^, ^45^ Recent research by Bonhoff et al. indicated that anxiety and depression might play a role in the association between CIPN and fatigue, further highlighting the complexity of the CIPN symptom burden.^46^ CIPN predominantly presents with sensory and sensorimotor impairments. The main symptoms of CIPN are pain, tingling, numbness, as well motor dysfunction such as difficulty buttoning, weakness, and falls. Psychological distress in patients with CIPN can be a direct result of these CIPN symptoms, but it may also moderate the presentation of these symptoms, for example, anxiety can impact how patients rate their pain and weakness. Thus, it is plausible that relieving psychological distress and subsequent coping mechanisms associated with CIPN might improve overall well‐being and quality of life.

To the best of our knowledge, this is the first yoga randomized controlled trial in gynecological and breast cancer survivors with persistent CIPN pain and one of the first to explore the effect of yoga on HRQOL outcomes in patients with moderate to severe CIPN. HRQOL outcomes are secondary endpoints in our study, and the results are exploratory and hypothesis generating. The study is also limited by the small sample size and lack of placebo control and long‐term follow‐up. The yoga arm had a higher drop‐out rate than usual care, which led to potential selection bias in the yoga arm as those who benefit from yoga are most likely to adhere to the intervention and follow‐up. In addition, the HADS anxiety scores were not balanced between yoga and wait‐list control arms, with lower anxiety in the wait‐list control arm at baseline, leaving very little room for improvement. Future clinical trials with larger sample size are needed to confirm our findings. In addition, further exploratory studies to elucidate yoga interventions and changes in serum biomarkers including BDNF, cortisol, and proinflammatory cytokines, as well as other biomarkers such as heart rate variability are also needed for cancer survivors with CIPN engage in yoga to improve psychological and physical well‐being.

## DISCLOSURES

All authors declare no conflicts of interest. We certify that there are no affiliations with or involvement in any organization or entity with any financial interest or other equity interests or non‐financial interests that influenced the design, outcome, and submission of this study.

## AUTHOR CONTRIBUTION

Conceptualization: W. Iris Zhi and Ting Bao. Methodology: Raymond Baser, W. Iris Zhi, and Ting Bao. Formal analysis: Raymond Baser and Qing S. Li. Acquisition of data: Dristi Talukder, Tina Paul, Clare Patterson, and Lauren Piulson. Writing—original draft preparation: Lillian M. Zhi, W. Iris Zhi, and Christina Seluzicki. Supervision, critical revision of the manuscript, and important intellectual input: W. Iris Zhi, Ting Bao, and Mary Lou Galantino.

## ETHICAL STATEMENT

The Institutional Review Board at Memorial Sloan Kettering Cancer Center approved this study.

## Data Availability

The data underlying this article will be shared at reasonable request to the corresponding author.
